# A Retrospective Study Regarding the Efficacy of Nuvola^®^ OP Clear Aligners in Maxillary Arch Expansion in Adult Patients

**DOI:** 10.3390/diagnostics15060738

**Published:** 2025-03-16

**Authors:** Sorana Maria Bucur, Radu Andrei Moga, Cristian Doru Olteanu, Eugen Silviu Bud, Alexandru Vlasa

**Affiliations:** 1 Department of Dentistry, Faculty of Medicine, Dimitrie Cantemir University of Târgu Mureș, 3-5 Bodoni Sandor Street, 540545 Târgu Mureș, Romania; bucursoranamaria@gmail.com; 2 Department of Cariology, Endodontics and Oral Pathology, School of Dental Medicine, Iuliu Hatieganu University of Medicine and Pharmacy, Motilor 33 Str., 400001 Cluj-Napoca, Romania; 3 Department of Orthodontics, School of Dental Medicine, Iuliu Hatieganu University of Medicine and Pharmacy, Avram Iancu 31 Str., 400083 Cluj-Napoca, Romania; 4 Department of Orthodontics and Dento-Facial Orthopedics, George Emil Palade University of Medicine, Pharmacy, Science, and Technology of Târgu Mureș, 38 Ghe. Marinescu Street, 540139 Târgu Mures, Romania; eugen.bud@umfst.ro; 5 Department of Periodontology and Oral-Dental Diagnosis, George Emil Palade University of Medicine, Pharmacy, Science, and Technology of Târgu Mureș, 38 Ghe. Marinescu Street, 540139 Târgu Mureș, Romania; alexandru.vlasa@umfst.ro

**Keywords:** Nuvola^®^ OP system, maxillary arch expansion, dental crowding correction, clear aligners

## Abstract

**Background/Objectives:** The study evaluated the effectiveness of Nuvola^®^ OP aligners, combined with an interceptive myofunctional device, in achieving dental arch expansions over an 18–26-month treatment period. **Methods:** 54 patients (31 women and 23 men, aged between 18 and 48 years old) participated in the study. The inclusion criteria for the present research were optimal oral hygiene, no prior orthodontic treatments, and no systemic conditions affecting outcomes of the treatment. Linear measurements (D1, D2, D3, D4) were obtained from STL files of the dental arches before and after treatment using Carestream CSMODEL™ software 3.10.47. Statistical analysis included MANOVA, Pearson’s correlation, and paired *t*-tests, following the Shapiro–Wilk test for data normality. **Results:** The treatment duration averaged 22.4 months. D1–D4 measurements means increased by 2.1 mm for D1, 2.37 mm for D2, 1.0 mm for D3, and 3.67 mm for D4. MANOVA results (*p* = 0.063) confirmed similar effects on all parameters, while Pearson’s correlation showed a weak positive association among distance changes. **Conclusions:** Nuvola^®^ OP aligners, used with an interceptive myofunctional device, effectively improved arch parameters. The significant increases in D1–D4 mean values suggest that this approach might be beneficial for controlled dental arch expansion in adult patients.

## 1. Introduction

Dental movements induced by aligners present unique challenges and complexities compared to those achieved with fixed appliances. These challenges arise from several factors, like the absence of specific application force points, variations in tooth anatomy, material properties, and discrepancies between the aligner’s geometry and the tooth’s surface [1,2]. To overcome these difficulties, Clear Aligner Therapy (CAT) employs a series of aligners designed to facilitate the gradual repositioning of teeth along small, preplanned trajectories [3,4].

The fundamental mechanism of clear aligners is based on the shape-molding effect, which directs targeted teeth to conform to the aligner’s shape [2,3,4]. This mechanism creates a three-dimensional (3D) force system that distributes pressure across all the contact surfaces of the tooth [2,4]. Recent advancements in numerical methods enable simulations of these forces, improving the predictability of treatment outcomes [5,6].

Tooth movement progresses through an aligner sequence, each with incremental adjustments that guide teeth from their initial positions to the desired alignment. This method proves effective for simple movements, such as minor rotations and slight positional changes [3,4].

A secondary mechanism relies on the ClinCheck^®^ Pro 6.0 software (Align Technology, Santa Clara, CA, USA) [7,8]. This software calculates the necessary movements for each tooth and adjusts the aligner’s shape accordingly. It incorporates pressure points to achieve vertical positioning and applies power forces for torque control, ensuring accurate force application during treatment [9].

Aligners must maintain consistent pressure throughout the treatment process. This pressure, applied through the shape-molding effect or additional attachments, is generally lower than that exerted by traditional brackets [10]. The pressure distribution is more extensive with aligners compared to the concentrated force of brackets. This broader distribution helps aligners retain their elastic properties over extended periods, enhancing their effectiveness [10,11].

CAT is commonly used to correct malocclusions in cases involving moderate crowding, interdental spacing, or relapses after fixed orthodontic treatments [12]. While additional attachments can expand the range of applications, limitations persist, including inadequate control over tooth movements and difficulties in achieving root rotations [13]. Overcorrections [14] and interproximal reduction (IPR) [15] may be necessary to optimize treatment outcomes.

Studies indicate that aligners efficiently control coronal tilt, but struggle with precise root movements. Specialized attachments can extend the range of movements, improving extrusion, rotation, and root tipping control [3,4]. Additionally, the effectiveness of aligner treatments depends on the precision of impressions, ideally obtained using intraoral scanners and simulation software [16].

Transparent aligners offer several advantages over fixed appliances, including reductions in the incidence of clinical emergencies, shorter treatment durations, enhanced aesthetics, increased comfort, and improved oral hygiene and periodontal health [17,18,19]. For adult patients with periodontal concerns, aligners contribute to better periodontal status and a reduction in bacterial levels, as demonstrated in a 12-month comparative study [20]. Aesthetic considerations further influence patients’ preference for clear aligners over traditional braces [21].

Although patients experience initial discomfort during the first week of treatment, the possibility of removing aligners offers greater functionality, psychosocial benefits, and reduced perceived pain compared to fixed appliances [21,22]. Treatment efficiency depends on thorough digital planning and the clinician’s expertise in leveraging available technologies [20].

The Nuvola^®^ OP System manufactured by Nuvola World Srl, Vicenza, Italy is an advanced orthodontic protocol that integrates traditional biomechanical principles of aligner therapy with functional orthodontic approaches. It requires precise diagnostic tools, including arch impressions, intraoral photographs, and radiographic examinations. Clinicians and technicians collaborate to develop a treatment plan, generating a 3D model that allows patients to visualize treatment objectives and limitations through digital simulations [21,22].

This system employs Nuvola^®^ OP aligners manufactured by Nuvola World Srl, Vicenza, Italy, and the Freedom™ myofunctional device manufactured by Myofunctional Research Co. (MRC), Helensvale, Australia [23]. The unique design of Nuvola^®^ OP aligners facilitates upper arch expansion through sectorial expansion zones that harness the tongue’s centrifugal forces. Proprioceptive stimuli help reprogram tongue posture within the oral cavity, maximizing its natural strength while minimizing dysfunctional movements [24].

The psychological impact of CAT is particularly significant among adult patients, as dissatisfaction with one’s smile can serve as a strong motivator for treatment [21]. However, sustained motivation requires continuous guidance and encouragement from orthodontic professionals [21].

Following the initial aligners set, progress assessments through new impressions help adjust the treatment plan, ensuring consistent accuracy. Custom attachments—tailored in location, shape, and thickness—enhance aligner retention and facilitate precise, planned movements. This level of control throughout treatment contributes to more predictable and satisfactory patient outcomes [23,24].

The Nuvola^®^ OP System’s combination of specialized aligners and myofunctional therapy expands treatment possibilities for complex cases by promoting harmony within the dental arches [25,26]. These aligners feature reinforced zones that stabilize specific tooth groups and influence palatal regions corresponding to sutures. By leveraging palatal morphology, the aligners address osteopathic lesions, using controlled muscle contractions to modulate sutures. Additionally, integrated lingual pins guide the tongue to the palate, improving swallowing function, influencing the premaxilla and occiput, and harmonizing temporalis muscle activity.

The retrospective study’ objective was to evaluate clinical efficacy in achieving the maxillary expansion of the Nuvola^®^ OP aligner system. Two key hypotheses were formulated to guide the investigation; the first hypothesis was that the Nuvola^®^ OP aligner system would not produce significant maxillary expansion. The second hypothesis was that patients with smaller baseline transverse dimensions would exhibit more expansion following treatment. These hypotheses provided a framework for assessing the aligner system’s effectiveness and identifying factors influencing treatment outcomes.

## 2. Materials and Methods

This study aimed to evaluate the effectiveness of treatment with Nuvola^®^ OP aligners in patients at our dentistry clinic. Patient recruitment was between June 2022 and September 2024. All participants provided written informed consent before inclusion. The study followed the guidelines of the Declaration of Helsinki and was approved by the Ethics Committee of SC Algocalm SRL, Târgu-Mureș, Romania (Approval No. 3/6 May 2022).

The sample size was determined based on prior literature [8,9,12,13,14], ensuring a statistical power (Pwr) of 80%, which resulted in a required sample of 49 patients. Ultimately, 54 patients, aged between 18 and 48 years, were enrolled in the study.

To minimize selection bias, patients were screened based on predefined inclusion and exclusion criteria. However, as participants were selected from a single clinical setting, the sample may not fully represent the broader population. Future studies should consider multi-center recruitment for improved generalizability.

The inclusion criteria within the research framework were as follows:

- Patients treated with the Nuvola^®^ OP System who had intraoral scans performed before and after treatment using a Carestream Intraoral Scan;
- Patients who wore the intercept myofunctional device during aligner treatment;
- Proper oral hygiene and good periodontal status;
- Healthy periodontium with no signs of inflammation.

The exclusion criteria were as follows:

- Patients with a previous history of orthodontic treatment;
- Signs of inflammation at the level of periodontal tissues;
- General disorders that could influence the treatment like diabetes mellitus or cardiovascular disease.

Linear measurements from STL files of the dental arches and expansion movements were analyzed before and after treatment using Carestream’s CSMODEL™ software. Measurements were recorded at baseline and post-treatment to assess changes.

Treatment methods:

The protocol required wearing the intercept myofunctional device for 30 min daily, alongside continuous wear of the aligners throughout the day.

The patient must have optimal periodontal health, since healthy gingival tissues ensure a perfect grip on the mouthpiece. All the investigated patients had professional oral hygiene 7–10 days before attachment application. The authors then informed the patients of the initial discomfort caused by the vestibular attachments, which would disappear within a few days. During the second appointment, tooth-colored attachments were placed according to the treatment plan to enhance aligner retention and guide the desired teeth movements. When receiving orthodontic devices, the patient received clear instructions on how to wear them and to behave properly. It was essential to wear the aligners 20–22 h a day. Regular check-ups were also crucial, to monitor the progress of the therapy, ensuring that the attachments were in good condition and that the aligners fit before moving on to the next set. Later, the authors completed a final check of the current aligner, and scheduled the next appointment. Before and after treatment, the authors registered—in a way very similar to that of the study conducted by Perrotti et al. [23]—the following parameters/measurements: D1 = the distance between the incisor papilla and the mesial-buccal cusp of the first molar in the right quadrant; D2 = distance between the incisor papilla and the mesial-buccal cusp of the first molar in the left quadrant; D3 = distance between canine cusps; D4 = the distance between the mesial-buccal cusps of the first molars.

**Figure 1:**
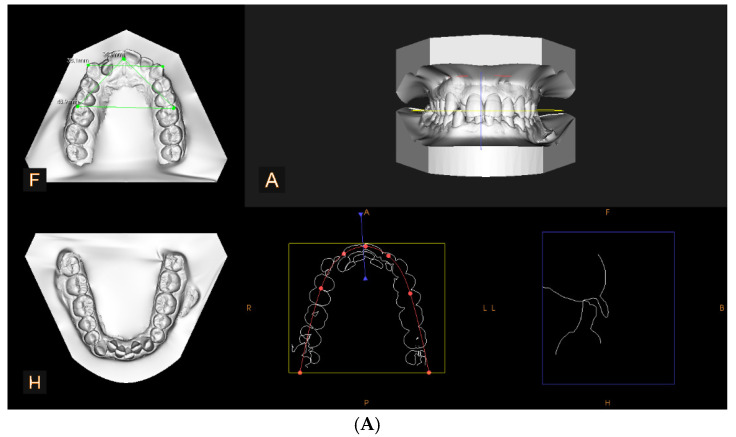

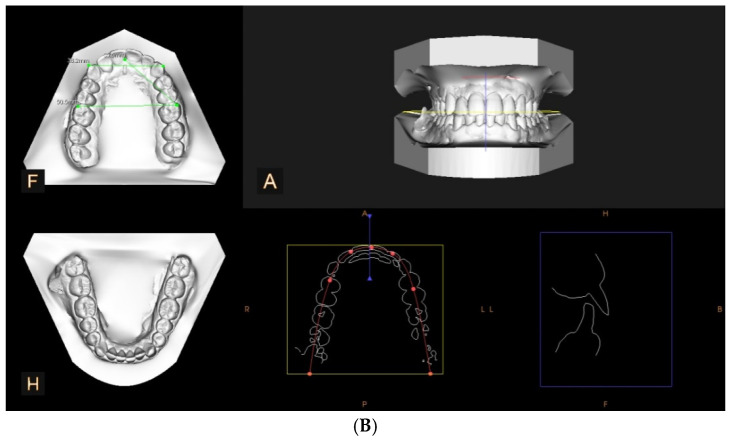
Cast model analysis: (**A**) initial aspect, D1 = 34.1 mm, D2 = 34.0 mm, D3 = 39.1 mm, D4 = 48.9 mm; (**B**) aspect after 22 months of treatment, D1 = 35.6 mm, D2 = 35.7 mm, D3 = 38.2 mm, D4 = 50.5 mm. (A) initial occlusal aspect of the upper and lower arches in centric occlusion; (F) occlusal view of the maxillary arch, including linear transverse measurements; (H) occlusal view of the mandibular arch; (R) right lateral aspect of the arch shape and alignment; (P) posterior view of the arch form and symmetry; (L) left lateral aspect of the arch shape and alignment; (B) frontal skeletal reference used for analysis.

Figure 1 shows the progression of a clinical case, both from the perspective of the D1–D4 parameters and from a frontal and lateral view.

Later in the study, the authors conducted simple descriptive and inferential statistics to analyze the changes in the four distances D1, D2, D3, and D4. For analysis, Multivariate Analysis of Variance (MANOVA) [27] was performed to evaluate whether the treatment had a uniform effect on D1, D2, D3, and D4, and Pearson’s correlation coefficient [28] was used to determine if changes in one distance are associated with changes in another. To determine the differences between D-parameters we used the paired *t*-test.

Before conducting Pearson’s correlation, the Shapiro–Wilk test for the normality of data were performed on the involved variables to ensure the validity of the analysis. The results of these tests confirmed that the variables followed a normal distribution, justifying the use of Pearson’s correlation.

A multiple linear regression analysis was conducted to evaluate factors influencing post-treatment distances. The model included age, gender, and baseline D1–D4 values as independent variables.

## 3. Results

The treatment duration ranged from 18 to 26 months, with an average duration of 22.4 months. The cohort consisted of 31 women (57.4%) and 23 men (42.6%). Age distribution was as follows: 16 patients (29.6%) were in the 18–28 age group, 25 patients (46.3%) were in the 28–38 age group, and 13 patients (24%) were in the 38–48 age group.

The MANOVA test suggested that the treatment similarly affected D1, D2, D3, and D4 (*p* = 0.063). The Pearson correlation analysis revealed weak to moderate positive associations (r = 0.28 to 0.46) among the measured distances (D1, D2, D3, and D4), suggesting a uniform impact of the treatment. Moderate correlations were observed between D1–D2 (r = 0.42), D1–D4 (r = 0.46), D2–D4 (r = 0.44), and D3–D4 (r = 0.39), indicating that changes in one distance were proportionally reflected in the others. Weaker correlations, such as D1–D3 (r = 0.28) and D2–D3 (r = 0.31), still suggest a consistent treatment effect. These findings support the notion that the Nuvola^®^ OP aligner system influenced all four distances comparably, reinforcing the uniformity of the intervention.

The Shapiro–Wilk test confirmed that the data followed a normal distribution (*p* > 0.05 for all parameters). Standard deviations (SDs) for the changes were as follows: D1: 0.35 mm (SD = 0.12 mm); D2: 0.41 mm (SD = 0.14 mm); D3: 0.28 mm (SD = 0.10 mm); D4: 0.47 mm (SD = 0.16 mm).

The arithmetic mean values of the pre-treatment measurements were as follows: D1 = 30.03 mm, D2 = 29.73 mm, D3 = 34.83 mm, D4 = 46.83 mm. Following the treatment, the arithmetic means of the values were observed to be as follows: D1 = 32.13 mm, D2 = 32.10 mm, D3 = 35.83 mm, and D4 = 50.50 mm. These values reflect the changes in measurements before and after the treatment, highlighting the impact of the applied intervention.

The multiple linear regression analysis assessed the influence of various factors on post-treatment distances, considering age, gender, and the baseline D1–D4 values as independent variables. The results indicated that age had a negligible impact on treatment outcomes (*p* = 0.215), while gender did not show a significant effect (*p* = 0.342). However, baseline distances emerged as a statistically significant predictor (*p* < 0.05), suggesting that patients with initially smaller distances experienced more expansion post-treatment. The regression model accounted for 68% of the variance in post-treatment measurements (R^2^ = 0.68), emphasizing the strong influence of baseline distances on outcomes.

The four investigated parameter averages (D1, D2, D3, and D4) revealed an increase in all four values after the treatment compared to initial values (Table 1).

## 4. Discussions

The results of this study indicate a significant and consistent increase in all measured parameters following treatment with the Nuvola^®^ OP aligners. Initially, the study hypothesized that the Nuvola^®^ OP aligner system would not produce significant maxillary expansion (H1). However, the data contradict this hypothesis, as all measured distances showed statistically significant increases (*p* < 0.05), rejecting H1. These findings also support the secondary hypothesis (H2), suggesting that patients with smaller baseline distances experience more post-treatment expansion.

In a cohort of 54 patients, the treatment produced a 2.1 mm increase in D1, reflecting expansion in the maxillary right quadrant. This aligns with the findings of Ravera et al. [29], confirming the success of treatment in this region. Similarly, D2 showed a 2.37 mm increase, indicating expansion in the maxillary left quadrant, consistent with previous studies [29]. Although the difference between D1 and D2 was not statistically significant (r = 0.42), the data suggest that these values reflect similar treatment effects in both quadrants. Notably, the slight difference observed in D1 may reflect a more severe pre-treatment condition in the right quadrant or a differential response to the treatment.

The treatment approach, which included composite attachment use, allowed distal movement of the maxillary first molars by 2.25 mm, with minimal tipping or vertical displacement. This approach did not alter facial height, as seen in the findings of Ravera et al. [29].

The 1 mm increase in D3, representing the distance between the canine cusps, reflects a transverse expansion of the anterior arch. This is consistent with findings by Ma et al. and D’Antò et al. [30,31], suggesting improved positioning of the canines and alignment of the anterior teeth, thus contributing to increased space. The maximum increase was observed in D4 (3.67 mm), indicating substantial expansion in the posterior arch between the first molars. This is consistent with similar findings from Ma et al. and D’Antò et al. [30,31] and reflects a significant improvement in occlusion and posterior arch alignment, which is crucial for functional and aesthetic outcomes [30,31,32].

In line with previous studies, this research demonstrates an increase in anteroposterior and transverse maxillary dimensions, facilitating dental alignment and improving occlusal function [30,31,32]. The expansion observed in intercanine and intermolar widths, as well as arch length, corresponds to increased space for optimal tooth alignment. These findings are consistent with the literature, which indicates that a 2–3 mm increase in intercanine and intermolar width typically enhances arch coordination, reduces crowding, and improves smile aesthetics [29].

While the study provides evidence of significant changes in arch dimensions, further investigation is needed to assess the clinical relevance of these changes. In orthodontics, arch expansion is crucial for enhancing occlusal function, stability, and aesthetics. However, the degree of change required for optimal treatment must be evaluated against established clinical standards. In this study, the increases in D1–D4 are within the clinically beneficial range reported in research on aligner therapy [29,30,31]. Furthermore, proper transverse arch development can improve facial harmony, bite function, and long-term periodontal health, potentially reducing the risk of relapse.

Although arch expansion with Invisalign^®^, manufactured by Align Technology, Inc., headquartered in Tempe, AZ, USA is not predictable, aligner therapy is still a practical option for addressing dental crowding. In another study, the effectiveness of expansion tends to be more noticeable in the premolar area [30]. When planning for arch expansion using Invisalign^®^, including overcorrection may also improve the treatment outcome. In the maxilla, the degree of expansion using Invisalign^®^ decreases from the front to the back, and setting appropriate buccal root torque for the posterior teeth may enhance the expansion [30], as also seen here. In our study, Nuvola^®^ OP aligners offered more predictable and consistent expansion across anterior and posterior segments.

A recent study on Ordoline aligners [31] found significant changes in maxillary transversal dimensions; however, the actual changes were less than the predictions. Significant differences were seen in all evaluated parameters, except for molar inclination, between the achieved movements and the prescribed values (*p* < 0.05). The study demonstrated an overall accuracy of 64% in the lower arch and 67% at the cusp level, while the gingival level exhibited a 59% accuracy. For the upper arch, total accuracy reached 67%, with 71% accuracy at the cusp level and 60% at the gingival level. Notably, the mean accuracy for molar inclination was significantly lower, averaging only 40%. Unlike our study’s results, the degree of expansion was higher at the canine cusps than at the premolars, with the least expansion being observed in the molars. Aligners primarily expand the arch by tipping the crown rather than moving the entire tooth, including the root, as the authors reported.

Enhanced aligner activation combined with additional force via a power arm increased the rate of movement for both the canine and the aligner as reported by Inan and Gonca [33]. They concluded that employing a power arm in canine distalization reduced distal tipping while increasing palatal tipping. This tool could minimize mesiodistal tipping in canine distalization during aligner treatment, though careful management was needed to control palatal tipping of the canine crown. As the power arm lengthened, von Mises’s stress [5,6,7] in the system increased, and the added activation and force raised principal stress levels within the periodontal ligament of the canine [34].

This effect is due to the reinforced morpho-corrector shields placed on the vestibular side of the alveolar process. These shields reduced the centripetal pressure exerted by the perioral muscles, particularly the buccinator muscle [23]. The result was the transverse development of the maxillary arch. The same principle applies to the shields of the Fränkel device [3]. This mechanism enhances the effectiveness of the planned expansion with the aligner and promotes the remodeling impact of the tongue on the alveolar process.

The Invisalign^®^ system, while effective in dental alignment, typically produces less predictable arch expansion. Studies have shown that Invisalign^®^ aligns the teeth through bodily translation and crown tipping, with a tendency for expansion at the cusp tips rather than at the gingival margins [35]. This approach often results in more buccal tipping of the posterior teeth rather than full-bodied expansion, which can be less efficient in achieving optimal transverse arch development. Moreover, the expansion achieved with Invisalign^®^ tends to diminish from the anterior to the posterior segments of the maxilla, as seen in studies where molar expansion is less than predicted by ClinCheck^®^ Pro 6.0 software. In contrast, the Nuvola^®^ OP system appears to offer a more consistent and effective expansion across both the anterior and posterior segments, with an overall increase in the transverse dimensions of the arch.

The Nuvola^®^ OP system stands out by applying intense, intermittent forces directed toward the cranial base, achieved through the activation of the intercept myofunctional device [36]. This device was engineered to promote skeletal adaptations in the cranial base by harnessing targeted myofunctional stimuli facilitated by movements that the patient independently performs for 30 min daily [23,36].

Although the daily 30 min use of the morpho-corrective device represents a relatively brief period, the technique shows promising results. It is especially noteworthy given that alveolar bone and teeth remain responsive to muscular forces throughout the rest of the day. The approach is metal-free, minimally invasive, and reduces the dependence on practitioner skill, making it a user-friendly option for cranial skeletal adaptation [23,36].

Traditional methods can yield similar results; however, they tend to be more invasive and costly due to the various laboratory products involved. The workforce in this sector is growing, leading to increased variability in outcomes influenced by the operators’ manual skills [37]. Moreover, the lingual pins on aligners play a crucial role in restoring the compromised lingual function in open bite malocclusions. These pins provide a continuous corrective stimulus 24 h a day throughout the treatment, ensuring effectiveness regardless of the patient’s self-discipline [38].

Bouchant et al. [39] examined the effectiveness of Invisalign^®^ treatment using invisible aligners for achieving transverse maxillary expansion. They found that this expansion primarily occurs in the dentoalveolar area, resulting in a tilting of the crowns of the posterior teeth, similar to our results regarding D3 and D4 parameter changes. Additionally, the Clincheck^®^ Pro 6.0 software tends to overestimate the extent of expansion movement compared to the actual clinical outcomes, leading to lower predictability of tooth movements, as also reported by D’Antò and Santucci [31,35].

Experience indicates that certain dental movements cannot be effectively achieved with aligners, although the limitations remain unclear [40], with our study being in agreement with that. Additionally, previewing the potential results may often mislead clinicians and patients. The effectiveness of transverse expansion of the maxillary arch is assessed at an average of 70% of prescriptions, which does not depend on the type of tooth and is applicable across the board [23,35], as herein clearly demonstrated. The efficacy between prescription and achievement is lower in the mandibular arch, with averages of 55% at the intermolar dimension and 46% at the canine level [31]. The present study found that expansion at the molar level was more than that at the canine level, consistent with previous findings [41]. In another study [30], the efficiency of corporal buccal expansion for maxillary first molars was, on average, 36.35%.

Statistically significant differences were identified between the effectiveness measured at the cusps and that measured at the most apical point of the palatal surface of the tooth [41]. This suggests more tipping movement than gingival movement. While the ClinCheck Pro 6.0 software programs movements for the tooth body, the results tend to reflect a tipping movement.

A study on the Invisalign^®^ system with SmartForce^®^ G8 technology, manufactured by Align Technology, Inc., headquartered in Tempe, AZ, USA demonstrates that clear aligners are effective for simultaneous intra-arch expansion in both the maxillary and mandibular arches [42]. Our research findings indicate that the expansion achieved with aligners is most effective in the premolar area, with lower efficiency in the canine and first molar regions. Expansion predictability was generally reasonable, though there were minor deviations in under-expansion and over-expansion [42].

Further analysis of treatments using SmartTrack^®^ aligners, manufactured by Align Technology, Inc., Tempe, AZ, USA supports these findings, highlighting that clear aligners can produce effective arch expansion. Like the SmartForce^®^ G8, the expansion effectiveness in SmartTrack^®^ treatments was highest in the premolar area and less in the canine and second molar regions [43].

Bruni et al. argued [44] that although clear aligners are used in dentistry, there is still much room for improvement in the predictability of tooth movements and subsequent bone development. The results here agree with other authors’ findings [30,35]. As research activities have been strengthened, substantial progress can be expected soon.

Zhou and Guo et al. [45] demonstrated that the preset amount of expansion movement and initial torque is negatively correlated with the efficiency of body expansion. Thus, it is necessary to preset sufficient torque on the buccal roots of the posterior teeth based on to the preset amount of expansion and initial torque.

Other authors [46] described the coronal tilt that prevails in the case of treatments with clear aligners, compared to corporal tooth migrations using an RPE—Rapid Palatal Expander. RME generally demonstrated better outcomes than Invisalign First Phase I treatment across all measured aspects. The only parameter with a statistically significant difference between the two treatments was the change in intermolar width at the gingival level, indicating that patients in the Invisalign First Phase I group experienced some buccal tipping [46]. The force produced by the RPE was roughly ten times stronger than the force generated by clear aligners [47].

Our study provided valuable insights into the benefits and challenges of early orthodontic treatment using aligners, particularly in addressing various malocclusion problems during the primary and mixed dentition phases, as reported by other studies [25,37,45,48].

As shown in previous studies [33,43], the intermolar distance experiences the largest increase during palatal expansion. In contrast, the intercanine distance exhibits the smallest increase, indicating that the anterior section of the maxillary arch has a higher resistance to expansion [31,42,43]. Additionally, another study on rapid maxillary expansion confirmed that through this expansion method, the intercanine area shows the lowest variation in transverse dimensions [49,50].

A study by D’Souza et al. [49] examined dental arch changes following rapid maxillary expansion (RME) and fixed orthodontic treatment, finding that arch width expansion corresponded to the increased arch perimeter, similar to our study, which demonstrated an increase in anteroposterior and transversal maxillary dimensions. Specifically, the intercanine width grew by 2.9 mm, the first premolar width by 3.2 mm, the second premolar width by 4.6 mm, and the molar width by 4.4 mm, while the arch perimeter increased by 3.2 mm, different from our findings, which showed that the intercanine width increased by 1 mm and the molar width grew by 3.67 mm. Strong positive correlations were found between arch perimeter and width increases at the canine, premolar, and molar regions. So, changes in intercanine and intermolar widths following palatal expansion, based on our study, may increase the arch circumference, which can help reduce the discrepancy between tooth size and arch length. Therefore, palatal expansion should be considered when addressing dento-maxillary disharmony associated with crowding. These findings support arch expansion as a non-extraction approach for borderline cases needing additional space [49].

However, when anterior crowding is present, it is advisable to incorporate a three-dimensional appliance to enhance changes in the intercanine dimension, as shown herein and in agreement with other studies [51,52].

The transparent nature of clear aligners makes them highly appealing aesthetically, which is particularly relevant for children concerned about facial appearance [48]. Nevertheless, the aligner treatment is not as painful as that of classic fixed appliances [53,54]. Our findings underscore the importance of evidence-based orthodontic decision-making and highlight the evolving paradigm of orthodontic care, with clear aligners playing a significant role in meeting patients’ growing needs. However, further research, particularly randomized controlled trials with larger sample sizes, is recommended to improve understanding and optimize treatment outcomes in early orthodontic intervention with clear aligners [48].

Other studies [25,48] exploring orthodontic expansion, Class II malocclusions, and the impact of aligners on gingival and periodontal health have provided further evidence of the efficacy and safety of aligner treatments in various orthodontic conditions. Clear aligners are effective for achieving desired dental aesthetics and can improve periodontal health compared to traditional fixed braces [55]. Furthermore, research evaluating the efficacy, safety, and acceptability of clear aligners during the primary and mixed dentition phases indicated levels of efficacy comparable to those of traditional fixed appliances, with potential advantages in treatment duration and patient comfort [12,17,22,48,52].

Baseline intercanine and intermolar distances were significant predictors of post-treatment expansion (*p* < 0.05). Patients with smaller initial widths experienced greater transverse changes, emphasizing the role of pre-treatment arch dimensions in predicting outcomes. This highlights the need for individualized expansion strategies based on baseline measurements. These findings align with previous research. One study found that for every millimeter of intercanine tip space deficiency expansion followed a predictable regression equation (y = 0.09 + 0.52x) [56]. Another long-term study confirmed that initial intercanine width predicts post-treatment dimensions, reinforcing the importance of baseline measurements [57].

This study provides valuable insights into the effectiveness of Nuvola^®^ OP aligners, particularly in combination with an interceptive myofunctional device, a relatively underexplored approach. The well-defined patient cohort, clear inclusion/exclusion criteria, and standardized measurement methods ensure robust data collection. The statistical analysis (MANOVA, Pearson’s correlation, and paired *t*-tests) strengthens the validity of the findings by identifying significant changes and correlations in treatment outcomes.

While this study provides valuable insights into the effectiveness of the Nuvola^®^ OP aligner system, it has several limitations. The absence of a control group restricts direct comparison with alternative orthodontic treatments, limiting the ability to assess the relative efficacy of the Nuvola^®^ OP system. Additionally, although the sample size was statistically powered, expanding it could improve the generalizability of the findings. Another key limitation is the lack of long-term follow-up, which prevents evaluation of post-treatment stability and long-term effectiveness.

To strengthen the evidence base for the Nuvola^®^ OP system’s clinical performance, future research should address these limitations by incorporating control groups like patients treated with traditional fixed appliances or other clear aligners, expanding the sample size, and conducting long-term follow-up studies.

Further investigation into the impact of baseline arch dimensions on post-treatment outcomes would provide a deeper understanding of the most effective use of the Nuvola^®^ OP aligner system across malocclusion types.

## 5. Conclusions

The Nuvola^®^ OP aligner system effectively achieves maxillary arch expansion and occlusion correction. Significant improvements in arch dimensions, particularly in the posterior region, highlight the system’s efficiency in widening the maxilla. The myofunctional device enhanced the occlusal outcome, providing better alignment and functional improvement. Based on these findings, clinicians should consider the Nuvola^®^ OP aligner system a reliable treatment option for patients seeking non-invasive solutions for arch expansion, especially those with smaller baseline distances. These results suggest the system’s potential for comprehensive orthodontic treatment, though further studies are needed to assess its long-term effects and broader applicability.

## Figures and Tables

**Table 1:** Summary of findings.

Parameter	Pre-Treatment (mm)	Post-Treatment (mm)	Change (mm)	Mean Change (mm)	Standard Deviation (mm)	*p*-Value (Paired *t*-Test)
D1	30.03	32.13	+2.10	2.1	0.35	<0.05
D2	29.73	32.10	+2.37	2.37	0.41	<0.05
D3	34.83	35.83	+1.00	1.0	0.28	<0.05
D4	46.83	50.50	+3.67	3.67	0.47	<0.05

## Data Availability

The original contributions presented in the study are included in the article; further inquiries can be directed to the corresponding authors.
